# The Use of Magnetoencephalography in the Diagnosis and Monitoring of Mild Traumatic Brain Injuries and Post-Concussion Syndrome

**DOI:** 10.3390/brainsci15020154

**Published:** 2025-02-04

**Authors:** Ioannis Mavroudis, Dimitrios Kazis, Foivos E. Petridis, Ioana-Miruna Balmus, Alin Ciobica

**Affiliations:** 1 Department of Neurosciences, Leeds Teaching Hospitals, Leeds LS9 7TF, UK; i.mavroudis@nhs.net; 2 Academy of Romanian Scientists, 050094 Bucharest, Romania; alin.ciobica@uaic.ro; 3 Third Department of Neurology, Aristotle University of Thessaloniki, 54124 Thessaloniki, Greece; dimitrios.kazis@gmail.com (D.K.); f_petridis83@yahoo.gr (F.E.P.); 4 Department of Exact Sciences and Natural Sciences, Institute of Interdisciplinary Research, “Alexandru Ioan Cuza” University of Iasi, 700057 Iasi, Romania; 5 CENEMED Platform for Interdisciplinary Research, University of Medicine and Pharmacy “Grigore T. Popa”, 700115 Iasi, Romania; 6 Department of Biology, “Alexandru Ioan Cuza” University of Iasi, 700505 Iasi, Romania; 7 “Ioan Haulica” Institute, Apollonia University, 700511 Iasi, Romania

**Keywords:** magnetoencephalography, mild traumatic brain injury, post-concussion syndrome, cognitive impairment, functional connectivity, brain oscillations, neuroimaging, machine learning, default mode network

## Abstract

**Background/Objectives**: The main objective of this systematic review was to explore the role of magnetoencephalography (MEG) in the diagnosis, assessment, and monitoring of mild traumatic brain injury (mTBI) and post-concussion syndrome (PCS). We aimed to evaluate the potential of some MEG biomarkers in detecting subtle brain abnormalities often missed by conventional imaging techniques. **Methods**: A systematic review was conducted using 25 studies that administered MEG to examine mTBI and PCS patients. The quality of the studies was assessed based on selection, comparability, and outcomes. Studies were analyzed for their methodology, evaluated parameters, and the clinical implications of using MEG for mTBI diagnosis. **Results:** MEG detected abnormal brain oscillations, including increased delta, theta, and gamma waves and disruptions in functional connectivity, particularly in the default mode and frontoparietal networks of patients suffering from mTBI. MEG consistently revealed abnormalities in mTBI patients even when structural imaging was normal. The use of MEG in monitoring recovery showed significant reductions in abnormal slow-wave activity corresponding to clinical improvements. Machine learning algorithms applied to MEG data demonstrated high sensitivity and specificity in distinguishing mTBI patients from healthy controls and predicting clinical outcomes. **Conclusions:** MEG provides a valuable diagnostic and prognostic tool for mTBI and PCS by identifying subtle neurophysiological abnormalities. The high temporal resolution and the ability to assess functional brain networks make MEG a promising complement to conventional imaging. Future research should focus on integrating MEG with other neuroimaging modalities and standardizing MEG protocols for clinical use.

## 1. Introduction

It is currently accepted that traumatic brain injury (TBI) is one of the most important causes of physical and mental disability, affecting millions each year. The severity of TBI can range from mild to severe, with mild TBI (mTBI) or concussion being the most common form, accounting for approximately 90% of all TBIs [1]. Despite its prevalence, mTBI uniquely challenges clinical diagnosis due to the often subtle and transient nature of its symptoms, as well as the limitations of conventional imaging techniques in detecting mild brain injuries following concussions [2]. Mild TBI patients frequently experience quality-of-life-affecting and persistent cognitive, emotional, and physical symptoms, conventionally referred to as post-concussion syndrome (PCS). Given the limitations of traditional imaging techniques in diagnosing mTBI and PCS, there is a growing need for more sensitive and objective tools to detect and monitor the underlying neural dysfunction associated with these conditions [3].

Magnetoencephalography (MEG) has emerged as a promising neuroimaging tool for mTBI and PCS diagnosis [4]. Unlike structural imaging techniques, MEG provides a functional assessment of brain activity by measuring the magnetic fields generated by neuronal currents. With its high temporal resolution, MEG can capture dynamic brain activity in real time, offering valuable insights into the neural mechanisms disrupted by brain injury. Recent advances in MEG technology coupled with machine learning approaches have allowed for a more precise identification of functional abnormalities in mTBI patients, even in cases where conventional imaging methods fail to detect any structural damage [4]. MEG has been used to investigate various neural changes associated with mTBI, including alterations in oscillatory brain activity, disruptions in functional connectivity, and abnormal slow-wave generation. Several studies have demonstrated that MEG can detect abnormal low-frequency activity, particularly in the delta and theta frequency bands, which often indicate neural injury [5,6,7,8,9]. Moreover, MEG has shown promise in identifying biomarkers for mTBI, which could assist diagnosis, provide insight into the mechanisms underlying persistent symptoms, and guide personalized treatment approaches. In some cases, post-traumatic stress disorder (PTSD) co-occurs or is confounded with mTBI and PCS, as some of the symptoms are overlapping [10]. As a consequence, it is important that the differentiated diagnosis is performed with accuracy and the patients receive proper care.

The aim of this systematic review is to assess the role of MEG in the diagnosis and monitoring of mTBI and PCS by evaluating the sensitivity of MEG in detecting functional brain abnormalities, comparing its effectiveness with traditional imaging modalities, and exploring its potential as a diagnostic tool for differentiating mTBI from other conditions with overlapping symptoms, such as PTSD. Additionally, the potential use of MEG as a monitor for the recovery of mTBI patients and the application of machine learning techniques in improving diagnostic accuracy will be pursued as secondary aims. In this way, the present study will make significant contributions regarding the utility of MEG in the clinical management of mTBI and the unmet diagnostic needs as a consequence of the heterogeneity of both cohorts that have previously been assessed and the type of analyses that have been employed during the assessments, albeit considering the novel data and recent advances in the field.

## 2. Materials and Methods

### 2.1. Study Design

**Figure 1:**
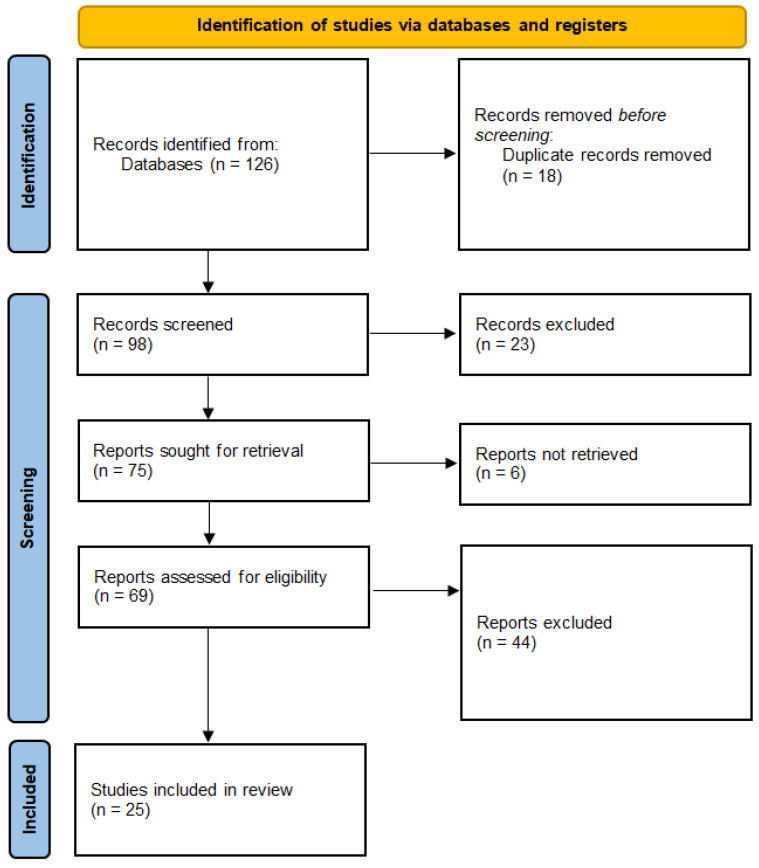
PRISMA flowchart.

This systematic review was conducted following the Preferred Reporting Items for Systematic Reviews and Meta-Analyses (PRISMA) guidelines and was registered to the PROSPERO platform (reg. no. CRD42024598973). The PRISMA flowchart could be found at Figure 1. This review aimed to evaluate the use of MEG in the diagnosis of mTBI and PCS, focusing on its ability to detect neural abnormalities, track recovery, and distinguish mTBI from other neuropsychiatric conditions.

### 2.2. Search Strategy

A comprehensive literature search was conducted across multiple electronic databases, including PubMed, Scopus, Web of Science, and Embase, to identify relevant studies published up until September 2024. The search terms included a combination of keywords and medical subject headings (MeSHs) such as “magnetoencephalography”, “MEG”, “mild traumatic brain injury”, “mTBI”, “concussion”, “post-concussion syndrome”, “PCS”, “functional connectivity”, and “brain oscillations”, separated by Boolean operators (e.g., “magnetoencephalography” OR “MEG” AND “concussion” OR “mild traumatic brain injury” OR “mTBI”; “mTBI” OR “PCS” AND “MEG” AND “functional connectivity”; “mTBI” OR “PCS” AND “MEG” AND “functional connectivity”; “mTBI” OR “PCS” AND “MEG” AND “brain oscillations”; and so on). The search was limited to English-written, human studies. Furthermore, their reference lists were searched for additional studies.

### 2.3. Studies Selection

#### 2.3.1. Inclusion Criteria

1. Population: Patients diagnosed with mTBI or PCS.
2. Intervention: Use of MEG for assessing brain function, either at rest or during cognitive tasks.
3. Outcomes: MEG-based biomarkers, such as abnormal oscillatory activity, functional connectivity disruptions, and slow-wave detection, in relation to diagnosing or monitoring mTBI or PCS.
4. Study Design: Observational (e.g., case–control and cohort studies) and experimental (e.g., clinical trials) designs.

#### 2.3.2. Exclusion Criteria

1. Studies not using MEG as the primary neuroimaging tool.
2. Studied not reporting results specific to mTBIs.
3. Review articles, meta-analyses, editorials, or opinion papers.

### 2.4. Data Extraction

The titles and abstracts of all retrieved articles were independently screened by two reviewers. Full texts of potentially relevant studies were reviewed for eligibility based on the inclusion and exclusion criteria, and for the selected studies, the following data were extracted:

- Study characteristics: author names, publication year, study design, and sample size.
- Participant characteristics: age, sex, and specific diagnosis (mTBI, PCS).
- MEG protocols: frequency bands analyzed (e.g., delta, theta, alpha, beta, gamma), resting-state versus task-based MEG, and analysis methods (e.g., functional connectivity, source localization).
- Outcomes: key MEG findings, including abnormal brain oscillations, connectivity changes, and correlations with cognitive or behavioral measures.

By its unique characteristics, MEG could detect the patterns of the five main types of brain wave oscillations as a result of synchronized neuronal activity. Each type of brain waves is associated with different physiological functions: delta (0.5–4 Hz)— sleep brain waves, which are increased in brain injuries and cognitive and attention deficits; theta (4 Hz)—deep relaxation brain waves, which are severely changed in insomnia; alpha (8–12 Hz)—awake, relaxed-state brain waves, which decrease with the occurrence of anxiety, high stress, and insomnia; beta (12–35 Hz)—active and attentive physiological-state brain waves, which are increased in anxiety and stress and decreased in attention deficit and depression; and gamma (>35 Hz)—concentration waves in which changes could suggest cognitive impairments [11,12,13,14,15].

A third reviewer provided additional evaluation when disagreements between reviewers occurred.

### 2.5. Data Synthesis and Analysis

Due to the heterogeneity of the studies in terms of study design, population, and MEG analysis methods, a quantitative meta-analysis was not feasible. Instead, a narrative synthesis of the findings was conducted. The results were organized into key themes, including the following:

1. MEG biomarkers for mTBI: Detection of abnormal oscillations (e.g., delta and theta waves) and their association with cognitive dysfunction.
2. MEG functional connectivity analysis: Disruptions in neural networks, particularly in the default mode network (DMN) and thalamocortical circuitry.
3. MEG for differentiating mTBI from PTSD: Studies that explored MEG’s ability to distinguish mTBI from PTSD based on distinct brain activity patterns.
4. MEG as a tool for tracking recovery: The role of MEG in monitoring changes in brain function over time and its correlation with clinical outcomes.

### 2.6. Quality Assessment

The quality of the included studies was assessed using the Newcastle–Ottawa Scale (NOS) for observational studies and the Cochrane Risk of Bias tool for clinical trials [16,17]. Each study was evaluated on standard criteria, such as sample representativeness, comparability between groups, and clarity of outcome measurement.

#### 2.6.1. Assessment of Observational Studies Using the Newcastle–Ottawa Scale (NOS)

The Newcastle–Ottawa Scale was used to evaluate the quality of observational studies based on three major domains: selection, comparability, and outcome. Each study was scored on a scale from 0 to 9, with higher scores indicating better methodological quality.

1. Selection of Participants. Most of the studies included in this review had well-defined inclusion criteria for patients with mTBI or PCS. Mainly participants from clinical settings were recruited, ensuring the representativeness of the target population. However, a few studies had small sample sizes, which may have affected the generalizability of their findings.
2. Comparability of Groups. Several studies included control groups of healthy participants (healthy controls, HCs) or individuals with orthopedic injuries (orthopedic trauma controls, OTCs) to allow for the comparison of MEG results. The studies that matched HCs based on age, sex, and education were rated highly for comparability. Some studies failed to control for confounding factors, such as co-occurring conditions—i.e., post-traumatic stress disorder (PTSD)—which could have introduced bias into the results.
3. Outcome Measurement. The outcome measurement for most studies was based on objective MEG biomarkers, such as abnormal oscillatory activity, functional connectivity, and slow-wave detection. Many studies used automated MEG analysis techniques, reducing the likelihood of observer bias. However, some studies relied on subjective cognitive assessments, which could have introduced variability in outcome reporting. A brief overview of the standard techniques for the analysis of MEG outcomes was previously addressed by multiple technical studies [15,18,19,20].

#### 2.6.2. Assessment of Randomized Controlled Trials Using the Cochrane Risk of Bias Tool

The Cochrane Risk of Bias tool was applied to the randomized controlled trials (RCTs) included in this review. This tool assesses the risk of bias across six domains: selection bias, performance bias, detection bias, attrition bias, reporting bias, and other potential biases.

1. Selection Bias. Randomization methods were generally well reported in the included RCTs. The majority of studies used appropriate randomization procedures, such as computer-generated random sequences, to assign participants to different intervention groups (e.g., MEG assessment versus other diagnostic tools). However, a few studies did not adequately describe their randomization process, leading to an unclear risk of selection bias.
2. Performance Bias. Blinding of participants and personnel was rarely implemented in the studies, particularly in those using MEG as a diagnostic tool. While blinding is challenging in diagnostic studies, the lack of blinding could have introduced performance bias in studies where subjective outcomes (such as cognitive performance) were measured alongside MEG data.
3. Detection Bias. Detection bias was minimized in most studies by the use of objective MEG biomarkers (e.g., frequency band analysis and source localization). Automated MEG analysis tools reduced the likelihood of bias in outcome detection. However, in studies that used cognitive tests as secondary outcomes, there was a potential for detection bias, especially if assessors were not blinded to the intervention groups.
4. Attrition Bias. Most studies reported low rates of participant dropout, and reasons for dropout were typically well documented. Studies with long follow-up periods, however, had higher rates of attrition, which could have influenced the results. The effect of attrition bias was generally low, as intention-to-treat analyses were applied in most cases.
5. Reporting Bias. Selective reporting of outcomes was minimal in the included studies, as most trials were pre-registered and reported all pre-specified outcomes. However, a few studies failed to report secondary outcomes, raising the potential for reporting bias.

### 2.7. Statistical Considerations

The characteristics of the included studies were summarized using descriptive statistics. A qualitative comparison of MEG results across different study designs and populations was conducted to highlight consistent patterns in the use of MEG for diagnosing mTBI and PCS.

## 3. Results

### 3.1. Summary of Quality Findings

#### 3.1.1. Overall Quality

The majority of the studies included in this review demonstrated moderate-to-high methodological quality. Most studies received favorable scores for participant selection and the use of objective MEG outcome measures, although some suffered from issues related to small sample sizes and lack of control for confounding factors.

#### 3.1.2. Key Strengths

○ The use of MEG as an objective high-resolution functional neuroimaging tool minimized observer and detection biases in many studies.
○ Studies that incorporated machine learning techniques in MEG analysis enhanced the precision of mTBI diagnosis and reduced subjective variability.

#### 3.1.3. Key Limitations

○ A lack of blinding in many studies and small sample sizes in some resulted in an increased risk of bias.
○ Studies that did not control for co-occurring conditions, such as PTSD or psychiatric disorders, may have confounded the findings related to mTBI and MEG biomarkers.

A total of 24 studies exploring the use of MEG in detecting functional and connectivity changes in mTBI, its correlation with cognitive impairments, and its potential as a biomarker for diagnosing mTBI and monitoring recovery were included.

### 3.2. MEG in mTBI Diagnosis and Biomarker Development

We found various studies [21,22,23,24,25,26,27,28,29,30,31,32,33,34,35,36,37,38,39,40,41,42,43,44] (Table 1) demonstrating the potential utility of MEG in mTBI and PCS diagnosis, particularly in detecting functional brain abnormalities that were not visible by conventional imaging techniques, such as MRI and CT scans. Moreover, more than one study conducted by Huang et al. [21,22,23,24,25,26] showed that MEG slow-wave abnormalities in delta (1–4 Hz) and theta (4–7 Hz) frequencies correlated with cognitive and emotional symptoms in mTBI patients, paving the way for identifying imaging biomarkers.

MEG also revealed abnormalities in white matter that were undetected by structural imaging. Alhourani et al. [27] and Rowland et al. [28] further supported the diagnostic utility of MEG by identifying disruptions in functional connectivity, particularly in the DMN. These disruptions were linked to long-term cognitive deficits in mTBI patients, highlighting the potential of MEG to uncover neural communication deficits that underlie PCS [27,28].

### 3.3. Functional Connectivity Changes in mTBI

MEG was found to be effective in detecting large-scale network changes in mTBI. Diwakar et al. [29] and Zhang et al. [31] demonstrated altered functional connectivity in frontal, temporal, and parietal regions in mTBI patients during resting-state and cognitive tasks. Specifically, MEG revealed connectivity-related changes in different brain regions, including the prefrontal cortex and medial temporal lobes. These changes were correlated with cognitive impairments, such as attention deficits and executive dysfunction. Particularly, Zhang et al. [31] concluded that beta wave patterns could predict cognitive dysregulations after mTBI.

Dunkley et al. [32] and Zhang et al. [33] reported beta-band oscillatory dysfunction in mTBI patients. Reduced beta-band power and connectivity were found in the thalamocortical circuits, which correlated with impairments in cognitive processing and motor control (Table 2).

### 3.4. MEG in Differentiating mTBI from PTSD

Several studies explored the role of MEG in differentiating mTBI from PTSD, a common comorbid condition (Table 3). Rowland et al. [28], Zhang et al. [33], and Popescu et al. [40] showed that MEG can differentiate between mTBI and PTSD based on differences in neural oscillations and network connectivity. MEG scans revealed distinct alterations in theta, alpha, and high gamma oscillations in the amygdala, hippocampus, and temporal regions, which were more pronounced in PTSD than mTBI.

These findings suggested that MEG may provide a valuable diagnostic tool for distinguishing between mTBI and PTSD, particularly when symptoms overlap, as conventional neuroimaging techniques often fail to differentiate between these conditions.

### 3.5. MEG as a Tool for Monitoring Recovery in mTBI

MEG has been shown to be a useful tool for monitoring recovery in mTBI patients (Table 4). Li et al. [34], Antonakakis et al. [36], and Lawton et al. [37] reported improvements in functional connectivity and reductions in abnormal slow-wave activity over time in mTBI patients, suggesting that MEG can track neural recovery. These studies demonstrated that MEG can detect improvements in brain connectivity patterns, which correlate with cognitive and behavioral recovery in mTBI patients.

Furthermore, Huang et al. [26] employed machine learning models to predict recovery based on MEG-derived neural activity. These models demonstrated high sensitivity and specificity in predicting mTBI outcomes, with improvements in functional connectivity correlating with better neuropsychological performance over time.

### 3.6. Cognitive and Behavioral Correlates of MEG Findings

Multiple studies linked MEG findings with cognitive and behavioral performance in mTBI patients (Table 5). Diwakar et al. [29] and Pang et al. [36] showed that alterations in brain connectivity detected by MEG were associated with deficits in mental flexibility, attentional control, and information processing speed. Patients with mTBI exhibited reduced functional connectivity in the prefrontal cortex and parietal regions, which correlated with impairments in executive functioning and processing speed.

In addition, Lawton et al. [37] demonstrated improvements in cognitive functions such as working memory, attention, and problem-solving skills following movement-based training, as detected by MEG.

### 3.7. MEG’s Role in Identifying Subtle Brain Injury

MEG’s ability to detect subtle brain injuries that are not visible on MRI or CT scans was highlighted in several studies (Table 6). Huang et al. [21] and Zhang et al. [33] demonstrated that MEG could reveal subtle changes in brain activity, particularly in the beta and gamma bands, which were associated with cognitive impairments in mTBI patients. Also, Rier et al. [39] described and compared the neuroimaging techniques with regard to the subtle brain injuries occurring as a result of mTBI concluding that MEG could detect small brain injuries with great accuracy and specificity, as compared to other neuroscans.

These findings suggest that MEG can detect brain dysfunction at a finer level of detail, providing valuable insights into the neurophysiological basis of mTBI.

### 3.8. Applications of Machine Learning and Deep Learning in MEG

Several studies applied machine learning algorithms to MEG data to improve diagnostic accuracy and predict recovery in mTBI patients (Table 7). Huang et al. [24] and Zhang et al. [33] employed machine learning models to classify mTBI patients based on MEG-derived connectivity patterns. These models achieved high diagnostic accuracy, sensitivity, and specificity, indicating that machine learning approaches can enhance the diagnostic capabilities of MEG.

## 4. Discussion

This systematic review aimed to highlight the significant role of MEG in the diagnosis and assessment of mTBI and PCS. MEG offers advanced neuroimaging scans with high temporal resolution, enabling the detection of subtle neural abnormalities that are often missed by traditional structural imaging modalities, such as MRI and CT scans. Thus, we explored a wide range of studies, each demonstrating MEG’s utility in identifying biomarkers for mTBI and PCS, particularly through measures of abnormal oscillatory activity and functional connectivity disruptions.

Since one of the first studies suggesting the possible use of MEG in detecting functional abnormalities of mTBI brain and providing wave patterns for mTBI by comparison to healthy controls [46], the interest for MEG in mTBI diagnosis significantly increased. Recently, a comprehensive review aimed at evaluating the potential of MEG detections in differentiating between mTBI and PTSD, yet the study concluded that the utility of MEG is mostly limited to research in TBI [47]. However, Allen et al [6] documented the use of MEG in mTBI diagnosis routines for adults by systematic review, but concluded that the data they collected had increased heterogeneity, thus not appropriate for meta-analyses. The efforts to improve the potential of MEG as a diagnosis and prognosis tool in mTBI were accompanied by complementing MEG with other powerful tools, such as other imaging scans (EEG, [48]), advanced cross-correlational analyses (synchronous neural interactions test [49]), or artificial intelligence (deep learning, Table 7). Further interest regarding MEG was paid not only for documenting differences in regional brain activation in mTBI, as compared to control brains, but also in timing of activation during relevant cognitive tasks [50]. In this context, the interest on MEG currently tends towards its possible use in detecting more subtle brain alterations, e.g. those resulted from sub-concussive impacts (head impacts not receiving a mTBI diagnosis) [51,52].

Overall, the results of this systematic review demonstrate that MEG is a powerful tool for diagnosing mTBI, monitoring recovery, and understanding the neurophysiological basis of PCS. MEG’s ability to detect subtle brain injuries, to identify functional connectivity changes, and to correlate with cognitive performance makes it a valuable addition to conventional neuroimaging techniques. However, further research is needed to standardize MEG protocols and validate its use in clinical practice.

### 4.1. MEG as a Diagnostic Tool for mTBI

Several studies provided strong evidence supporting the use of MEG in diagnosing mTBI, particularly through the detection of abnormal slow-wave activity. Huang et al. [21] identified abnormal low-frequency magnetic activity (1–4 Hz) in mTBI patients, which was linked to underlying axonal injury, as detected by DTI. This finding aligns with the results from other studies that observed increased delta and theta waves activity in mTBI patients, further demonstrating the sensitivity of MEG to pathological changes in brain activity that are not captured by conventional imaging techniques.

The ability of MEG to detect functional disruptions in specific brain regions also shows promising potential in identifying cognitive impairments associated with mTBI. Hung et al. [41] and Zhang et al. [33] reported significant increases in delta and theta oscillatory activities in frontal and temporal regions correlated with cognitive deficits in attention and executive function. Similarly, Popescu et al. [40] found that reduced alpha-band power in prefrontal regions was linked to the severity of PTSD symptoms in individuals with mTBI.

Moreover, recent relevant data suggested that newly developed artificial intelligence tools (machine learning) could be successfully used in increasing the potential of MEG as a diagnosis tool in mTBI. Thus, in some of the studies that were included in this systematic review, it was demonstrated that the classification of mTBIs and recovery prediction based on connectivity patterns analysis pipelines were improved [24,26,31,33,39,40,44]. In this context, Aaltonen et al. [53] compared three different machine learning-assisted algorithms for diagnosis accuracy and found that their performance was higher than the traditional evaluation of resting-state MEG power spectra in patients that were affected by mTBI at less than 2 months before the scan. It is important to note that machine learning-assisted evaluation of MEG spectra could be used a diagnosis tool in both adults and children, as seen in the studies we evaluated in this report. However, there could be differences between the MEG spectra by function of age and time from injury in mTBI patients, as suggested by some reports. Moreover, Safar et al. [54] suggested that machine learning-assisted evaluation of MEG spectra could also be employed in detecting neural functioning disturbances in chronic TBI and prolonged post-concussive syndrome, but they draw attention to the differences they observed in children and adolescents affected by chronic TBI—theta and gamma bands changes, subtle delta band changes, and alpha band peaks—in contrast to the discussed changes seen in adults.

### 4.2. Functional Connectivity and Network Disruptions

Beyond the identification of abnormal brain oscillations, MEG has proven effective in analyzing functional connectivity, particularly in mTBI patients. Functional connectivity disruptions, especially in the DMN and frontoparietal networks, were consistently reported across multiple studies. Alhourani et al. [27] used MEG to reveal significant reductions in functional connectivity across cortical regions involved in the DMN, suggesting that disruptions in this network may underline the cognitive and attentional deficits commonly observed in mTBI patients. Furthermore, Zhang et al. [33] showed that MEG could differentiate mTBI from other conditions, such as PTSD, by revealing distinct patterns of connectivity in specific brain regions.

Graph theoretical analyses of functional connectivity have also been useful in identifying network-level disruptions in mTBI patients. Kaltiainen et al. [42,43] and Itallina et al. [44] demonstrated that individuals with mTBI exhibited reduced local efficiency in brain networks, which is indicative of altered information processing. This supports the hypothesis that mTBI leads to widespread disruptions in functional networks contributing to cognitive and behavioral symptoms.

### 4.3. MEG as a Tool for Monitoring Recovery

In addition to its diagnostic capabilities, MEG has shown potential as a tool for monitoring recovery from mTBI. Several longitudinal studies included in this review reported improvements in MEG-derived biomarkers over time, paralleling clinical recovery. Huang et al. [23] found that reductions in abnormal slow-wave activity measured by MEG were associated with improvements in cognitive performance. These findings suggested that MEG could be used to track neural recovery and assess the effectiveness of interventions in mTBI patients. Moreover, studies have explored the use of MEG in predicting long-term outcomes in mTBI patients. Vakorin et al. [45] employed machine learning techniques to analyze MEG connectivity patterns and reported high-accuracy predictions of the severity of PCS. This highlights the potential for MEG to inform clinical decisions regarding prognosis and rehabilitation strategies.

### 4.4. Limitations of MEG in mTBI Diagnosis

Despite the promising findings, some limitations of MEG should be considered (Table 8). While MEG offers high temporal resolution, its spatial resolution is lower compared to other imaging techniques, such as functional MRI. This limitation could affect the precision of localizing brain regions involved in mTBI-related abnormalities. Furthermore, MEG is a relatively costly and less accessible neuroimaging technique, limiting its use in clinical practice. However, some of these limitations are currently addressed while developing new improved MEG systems, such as optically pumped magnetometer-assisted MEG, which enables the possible development of point-of-care testing systems with less expensive use and maintenance [55].

However, there could be some limitations regarding the use of MEG in mTBI diagnosis taking into account the demographic and clinical characteristics of the patients. Several studies have demonstrated that brain wave patterns could vary depending on physiological states (i.e., age, sex) [56,57,58,59] and in mTBI [60]. In this context, the studies we included in this analysis presented reports of brain activity in both children and adults. The findings suggested several differences regarding the variability of changes in brain wave patterns, concerning the wave types and the brain areas affected (Table 1). However, these differences could also be the results of the varied mechanisms of injury, as [25] demonstrated by comparing blast and non-blast mTBI cases for differences in brain activity. In this context, according to the included studies, most of the children/adolescents suffered from mTBIs that were due to falls or sports accidents, while the origin of injury in adults was more heterogeneous. Moreover, we observed that many studies focused on evaluating the changes in brain waves activity in males, rather than in females. This could be a result of the increased prevalence of concussions in men, as compared to women [61]. Further studies should focus on analyzing the possible differences that occur in mTBI cases based on age, sex, mechanism of injury, and other variables that could be implicated in brain activity variability, as demonstrated by several studies [62,63,64,65] for sex and age differences after mTBI in behavior and brain anatomy.

Moreover, the heterogeneity in study designs, MEG scan processing methods, and patient populations posed a challenge in comparing results across studies. Future research should aim to standardize MEG protocols for mTBI assessment to facilitate cross-study comparisons and improve the reliability of MEG as a diagnostic tool.

### 4.5. Implications for Clinical Practice and Future Perspectives

The results of this review demonstrate the potential of MEG as a valuable tool in the diagnosis and monitoring of mTBI and PCS. By providing objective biomarkers of brain function, MEG could complement traditional imaging modalities, which often fail to detect the subtle neural changes associated with mTBI. Furthermore, the use of machine learning techniques in conjunction with MEG data shows promise in improving diagnostic accuracy and predicting clinical outcomes. For instance, to overcome the limitation of lower spatial resolution, as compared to other imaging techniques, Antonakakis et al. [48] described a possible combination between MEG and EEG and analyzed the possibility to improve connectivity mapping by adding MRI with diffusion tensor imaging.

Future perspectives that are opened by this study (Table 9) include the necessity for larger, multi-centered studies that could enable the validation of the previous findings. Additionally, further exploration of MEG’s potential to differentiate between mTBI and co-occurring conditions, such as PTSD, could improve diagnostic precision and patient management. Developing more cost-effective and accessible MEG technologies will also be essential for translating these research findings into clinical practice.

### 4.6. Limitations of the Current Systematic Review

Except for the limitations of MEG as a diagnosis tool in mTBI, as presented in the selected studies and discussed above, this study has several limitations. For instance, the studies that were evaluated in accordance with the selection criteria reported a small number of patients and healthy controls with varying demographic characteristics (mainly age and mTBI source). In this context, further studies could concentrate on large-scale evaluations of mTBI patients and populations in order to provide more comprehensive results. Another important limitation was the heterogeneity of the parameters used in MEG evaluation, as well as in filtering the imaging data for noise, signal power, and connectivity type. However, this study was unable to perform a meta-analysis of the data, similarly to a previous report by Allen et al. [6], which failed to demonstrate the use of MEG in mTBI diagnosis routines for adults. Further meta-analyses could be performed only after a rigorous filtering of the collected data and by eliminating as many variables as possible.

## 5. Conclusions

MEG is a promising tool for the diagnosis and monitoring of mTBI and PCS. Its ability to detect abnormal brain oscillations and functional connectivity disruptions offers unique insights into the neural mechanisms underlying mTBI-associated brain damage and its long-term consequences. Significant reports endorsed the potential of MEG to assist in diagnosis and prognosis. The sensitivity of detecting abnormal brain oscillations and functional connectivity disruptions could provide significant advantages in understanding the subtle neural impairments often missed by traditional imaging methods. Also, several studies have demonstrated the correlation between MEG biomarkers and cognitive and behavioral symptoms that could accommodate differentiated diagnosis—between mTBI and overlapping or co-occurring conditions—and monitoring recovery. Despite its limitations, mainly concerning standardization and accessibility, our analysis showed that MEG could play a crucial role in improving the diagnosis, treatment, and prognosis of mTBI.

## Figures and Tables

**Table 1:** Studies focused on MEG for mTBI diagnosis and biomarker development.

Ref	Aim	Type	Design	Intervention	TIA	Outcomes	Conclusion
[21]	Evaluating the potential of MEG and DTI integrated imaging in detecting mTBI	OS	10 post-acute mTBI (aged 25.0 ± 11.5, 1 female soccer player, 3 male sport players, 4 male veterans, 2 males who had suffered motor vehicle accidents); 14 HCs (aged 27.4 ± 15.2)	Resting-state MEG while awake, eyes opened; frequency bands: delta waves (1–4 Hz); analysis methods: functional connectivity, source localization with VESTAL.	1–46 months	Subtle neuronal injury detected by MEG, not visible by CT or MRI; abnormal delta waves detected by MEG (in many brain areas, such as dorsal lateral prefrontal cortex, middle frontal gyrus, orbital frontal cortex, anterior cingulate cortex, and parietal–temporal junction regions), not seen by DTI.	MEG is more sensitive than traditional imaging techniques for diagnosing mTBI.
[22]	Evaluating task-related MEG in detecting functional deficits in mTBI	OS	25 male veterans with history of mTBI due to blasts or military training; 20 male veterans (HCs)	Task-related MEG (N-back WM task); frequency bands: alpha (8–12 Hz), beta (15–30 Hz), gamma (30–90 Hz), and low-frequency (1–7 Hz); analysis methods: functional connectivity, source localization with fast-VESTAL.	~10 months	Functional changes across several brain areas (increased signals in frequency bands across frontal pole, ventromedial prefrontal cortex, orbitofrontal cortex, and anterior dorsolateral prefrontal cortex; decreased signals in anterior cingulate cortex) correlated with slower reaction times in the working memory task.	Task-related MEG could evaluate functional deficits correlated with cognitive impairments.
[23]	Evaluating resting-state MEG as a potential diagnosis tool in pediatric mTBI patients	OS	24 children aged 8–15 years: 12 mTBI due to closed head injury (83% males, 8 falls, 3 head collisions, 1 motor vehicle accident); 12 HC or OTC (92% males)	Resting-state MEG, 3 × 5′ eyes-closed sessions alternating with 3 × 5′ eyes opened sessions; frequency bands: alpha band (8–12 Hz), beta band (15–30 Hz), gamma band (30–80 Hz), high-gamma band (80–150 Hz), and—combined delta (1–4 Hz) and theta bands (4–7 Hz); analysis methods: functional connectivity, source localization with fast-VESTAL.	~6 months	Changes in alpha (hyperactivation—prefrontal cortex, hypoactivation—parahippocampus and insula), beta, gamma (frontal, temporal, occipital, and cerebella regions), and low-frequency bands of mTBI patients during resting-state scanning; differences between adult and children mTBI scan patterns could occur.	Resting-state MEG could be used to discriminate between mTBI and non-mTBI children.
[24]	Evaluating resting-state MEG gamma bands as a potential biomarker for mTBI	OS	60 males active/retired military personnel: 25 mTBI due to blasts (aged 28 ± 7.52 years); 35 HCs (aged 29 ± 5 years).	Resting-state MEG, 2 × 5′ sessions with eyes closed, 2 × 5′ sessions with eyes open; frequency bands: gamma waves (30–80 Hz); analysis methods: functional connectivity, source localization with fast-VESTAL.	19.5 months	Significant changes in gamma band activity of mTBI patients, as compared to HC during resting-state scanning: hyperactivation in prefrontal cortex, supplementary motor area and right premotor cortex, posterior parietal areas, superior temporal gyri, occipital areas, right cerebellum.	Gamma waves activity evaluated by resting-state MEG could be a promising biomarker of mTBI.
[25]	Evaluating the potential of automated MEG in diagnosing mTBI	RCT	55 mTBI patients: 23 male military personnel (blast injuries, aged 26.0 ± 5.3 years); 22 civilians (mTBIs diverse injuries, aged 29.1 ± 13.3 years, 68% males); 10 civilians (moderate TBI diverse injuries, aged 29.2 ± 13.2 years, 80% males); 44 HC (aged 26.5 ± 8.0 years, 84% males).	Resting-state MEG; Frequency bands: delta waves (1–4 Hz); analysis methods: functional connectivity, source localization with frequency domain VESTAL.	4 weeks–3 years	Abnormal delta waves activity was found in 87% of mTBIs and 100% of moderate TBIs in 6 to 8 cortical regions; delta waves activity evaluated via resting-state MEG could discriminate between different sources of mTBI (i.e., blast versus non-blast); A correlation was established between abnormal slow-wave positive brain regions and PCS scores.	MEG low-frequency source imaging could be used to support the clinical diagnosis of TBI and PCS. MEG could perform significantly better in finding low-frequency waves changes in contrast to conventional neuroimaging approaches.
[26]	Evaluating the potential of automated resting-state MEG in diagnosing pediatric mTBI	LS	59 mTBI pediatric patients, aged 12.05 ± 2.22 years, 66.1% males; 39 OTC pediatric patients (upper or lower limb fractures, aged 11.18 ± 1.88 years, 64.1% males)	Resting-state MEG, 2 × 5′ sessions, eyes closed; frequency bands: delta (1–4 Hz) and gamma (30–80 Hz) bands; analysis methods: functional connectivity, source localization with fast-VESTAL.	3 weeks; 7 weeks; 12 weeks.	Changes were found for delta and gamma bands in frontal and temporal lobes: increased right and left frontal pole gamma band frequency—hypothesized mTBI imaging marker; resting state-MEG with machine learning successfully discriminated between mTBI and OTC.	Resting-state MEG could be used to support the clinical diagnosis of mTBI.
[27]	Detecting functional connectivity network disruption in mTBI	OS	9 mTBI patients, 4 females, aged 14–62 years; 15 HCs	Resting-state MEG, 2–5′ sessions with eyes opened; frequency bands: delta (1–4 Hz), theta (4–8 Hz), alpha (8–12 Hz), and beta (12–30 Hz) bands; analysis methods: functional connectivity with phase locking value.	3 months–3 years	Significant changes in brain activity were reported for delta, alpha, and beta frequency bands in superior parietal, cuneus, precuneus, inferior parietal, lateral occipital, and supramarginal lobules, with prominent beta phase locking reduction in the temporal lobes of mTBI patients.	MEG could detect brain activity changes in multiple frequency bands in mTBI.
[28]	Network disruption in mTBI	OS	6 mTBI patients (male veterans, aged 39.0 ± 9.5 years); 10 HCs	Resting-state MEG, 8′ session eyes opened; frequency bands: delta (0.5–4 Hz), theta (4–8 Hz), alpha (8–13 Hz), beta (13–30 Hz), gamma (30–80 Hz), as well as DC-80 Hz bands; analysis methods: functional connectivity between nodes using weighted phase lag index.	6.2 years	Resting-state MEG revealed that mTBI patients are not characterized by changes in alpha network metrics but by increased small worldness in the wideband network.	Graph-based analysis of resting-state MEG scans could be a valuable tool in diagnosing mTBI and co-morbidities.
[29]	Anticipatory control of eye movements in chronic mTBI	RCT	25 mTBI + PCS patients (aged 32.7 ± 11.24 years, 84% males, 4 motor vehicle accidents, 13 sport-related injuries, 5 falls and head blows); 25 HCs (aged 31.8 ± 10.6, 68% males)	Task-related MEG, 3 eyes movement task sessions; frequency bands: alpha (8–13 Hz), beta (15–30 Hz), and gamma (30–100 Hz) bands; analysis methods: functional connectivity, source localization with fast-VESTAL.	~32 months post-injury	Mild TBI patients performed poorer in the eye movement control task using gapped trajectories, as compared to HCs, and were correlated with beta band changes in ten brain regions.	Beta bands amplitude, as evaluated by MEG, could be used as a potent differentiator between mTBI and HCs.
[30]	Slow-wave detection in mTBI	RCT	31 mTBI + PCS patients (veterans, aged 26.6 ± 6.1 years, 90.3% males); 33 HCs (veterans, aged 26.3 ± 8.3, 93.9% males)	Resting-state MEG, 3 × 4′ eyes closed sessions; frequency bands: delta bands (1–6 Hz); analysis methods: functional connectivity, source localization with fast-VESTAL.	~3 months	Abnormal MEG slow-wave activity correlates with cognitive impairments (increased brain waves in prefrontal cortex and temporal areas; decreased brain waves in occipital area).	MEG provides objective evidence of brain injury in mTBI with PCS patients.
[31]	Exploring the beta waves changes in mTBI male patients	RCT	27 mTBI male patients (aged 29.6 ± 6.7 years, 12 sports injuries, 7 motor vehicle accidents, 4 falls, 4 other injuries); 23 male HCs (aged 28.0 ± 5.6 years).	Resting-state MEG, 1 × 5′ eyes-open session; frequency bands: alpha (8–14 Hz), beta band (15–30 Hz); analysis methods: functional connectivity, Fitting Oscillations and One-Over F (FOOOF) algorithm.	<3 months	Thalamocortical dysconnectivity was identified in mTBI patients. Alpha peaks were seen in mTBI visual cortexes. Reduced beta band frequency was seen in bilateral frontal and temporal cortices of mTBI patients, with the lowest negative peak in the right middle frontal gyrus. Beta band functional connectivity could be used to classify biomarkers for mTBI, with better performance as compared to SCAT2.	Beta band functional connectivity assessed by MEG could be used to diagnose mTBI.
[32]	Exploring the functional connectivity status in mTBI	OS	20 mTBI (males, 31.4 ± 6.85 years, 7 sports injuries, 7 motor vehicle accidents, 4 falls, 2 other injuries); 21 HCs (males, aged 27.0 ± 5 years)	Resting-state MEG, eyes opened; frequency bands: delta (1–4 Hz), theta (4–7 Hz), alpha (8–14 Hz), beta (15–30 Hz), low gamma (30–80 Hz) and high gamma (80–150 Hz); analysis methods: functional connectivity.	<3 months	Low-frequency functional connectivity affected in mTBI. Alpha bands reduced in parietal regions of mTBI affected brains; delta and theta band amplitudes increases were seen in the left hemisphere, left frontal, left temporal, and subcortical regions, and right posterior cingulate cortex of the mTBI-affected brains.	Low-frequency functional connectivity makers could be used in diagnosing mTBI.
[33]	Exploring the potential of neural oscillations to differentiate mTBI from PTSD	OS	27 mTBI (military personnel, aged 29.6 ± 6.7 years; 23 HCs (civilians, aged 28.0 ± 5.6 years);	Resting-state MEG, 1 × 5′ eyes opened session, fixed point; frequency bands: delta (1–3 Hz), theta (4–7 Hz), alpha (8–14 Hz), beta (15–30 Hz), low gamma one (30–55 Hz), low gamma two (65–80 Hz), and high gamma (80–150 Hz); analysis methods: functional connectivity.	<3 months	The regional power from alpha and beta bands could be used as diagnosis markers for mTBI.	MEG could be used to detect subtle brain functional connectivity changes in mTBI.
[34]	Exploring the potential of MEG to assess recovery from mTBI	LS	13 mTBI patients (aged 25.6 years, 54% males); 8 OTC (aged 27.2 years, 50% males).	Resting-state MEG, 1 × 5′ eyes-closed session; frequency bands: 0.1–4 Hz (delta), 4–8 Hz (theta), 8–10.5 Hz (alpha1), 10.5–13 Hz (alpha2), 13–20 Hz (beta1), 20–30 Hz (beta2), 30–40 Hz (gamma1), and 40–80 Hz (gamma2); analysis methods: source power analysis, BEM (boundary element method), functional connectivity.	Not reported	Significant right entorhinal and the left supramarginal gyrus in-flow connectivity in mTBI. Hyperactivity of delta band was seen in mTBI-affected brains (right isthmus cingulate, left pars triangularis, right postcentral, right precentral, and left precuneus). Stronger delta connections were seen in mTBI patients, as compared to OTC.	MEG could be used as a diagnosis tool for mTBI, with the delta band as a potential imagining biomarker.
[35]	Exploring the potential of MEG to detect brain connectivity alterations in mTBI	RCT	16 mTBI male patients (civilians with motor vehicle and sports accidents, aged 31.0 ± 7.5 years); 16 male HCs (aged 27.7 ± 5.3 years)	Task-related MEG, tasked session of 10–30′; frequency bands: theta (4–7 Hz), alpha (8–14 Hz), beta (15–30 Hz), and gamma (30–80 Hz); analysis methods: functional connectivity.	<2 months	Brain connectivity was reduced in mTBI, as compared to HCs, when evaluated by task-related MEG. Lower connectivity was seen for alpha band in the brains of mTBI patients, in occipital cortices.	MEG could be used to detect brain connectivity issues in mTBI.
[36]	Exploring the potential of resting state MEG to predict the occurrence of mTBI	OS	30 mTBI patients (aged 29.33 ± 9.2 years); 50 HCs (aged 29.25 ± 9.1 years).	Resting-state MEG, 1 × 3–5′ eyes-closed session; frequency bands: delta (0.5–4 Hz), beta (15–30 Hz) and gamma1 (30– 45 Hz); analysis methods: time-varying cross-frequency coupling.	<24 h	Dynamic network analysis showed microstate-related transitions in mTBI, as compared to HCs.	MEG could be employed to diagnose mTBI by identifying a dynamic connectome biomarker based on functional integration.
[37]	Exploring the potential of task-related MEG to detect cognitive remediation in mTBI	RCT	4 mTBI male patients (aged 15, 50, 62, and 68 years)	Task-related MEG (N-back WM task); frequency bands: beta band (15–30 Hz); analysis methods: sensor-waveform covariance matrix, source localization with fast-VESTAL.	Not reported	MEG brain imaging successfully detected changes in activity of several brain networks. Signal increases were seen in the prefrontal cortex, orbitofrontal cortex, ventrolateral prefrontal cortex, medial posterior parietal cortex, premotor cortex, and dorsal cingulate/medial premotor cortex.	MEG brain imaging could be used to detect brain network activity mTBI.
[38]	Exploring the potential of MEG to detect functional connectivity alterations and correlations with visual attention and working memory in mTBI	RCT	18 mTBI male patients (aged 30 ± 7.3 years, 6 sports injuries, 4 falls, 4 motor vehicle accidents, 3 other accidents, and 1 work-related injury); 19 male HCs (aged 27 ± 5.2 years)	Task-related MEG (visual working memory 1-back task); frequency bands: multiple (1–50 Hz); analysis methods: functional connectivity, source localization with fast-VESTAL.	1.2 months	MEG was able to detect abnormal brain waves patterns in concussed patients (increased in left lingual gyrus and right hippocampus; decreased in right superior parietal, inferior parietal, and the left middle frontal cortices), correlated with visual working memory processing, attention, processing, and retrieval. The changes in frequency bands were correlated with symptom severity.	MEG could be used to discriminate between mTBI and HCs based on brain activity and cognitive tasks.
[39]	Evaluating the potential of MEG-related functional connectivity as a diagnosis tool for mTBI	OS	29 male mTBI patients; 23 male HCs	Resting-state MEG, 1 × 5′ eyes-opened session and motor-task MEG; frequency bands: multiple; analysis methods: functional connectivity, source localization with hidden Markov model.	<3 months	By using MEG in resting state and during motor tasks, it was shown that mTBI disrupts the structural framework underlying neural synchrony and network functions, which are both very subtle consequences of head trauma.	Subtle changes in brain connectivity could be assessed by resting-state and task-related MEG.
[40]	Evaluating alpha-band power in mTBI with PTSD	OS	32 male mTBI patients (active-duty service members)	Resting-state MEG, 1 × 5′ closed-eyes session; frequency bands: alpha band (8–13 Hz); analysis methods: functional connectivity, source localization.	6 months to 11 years	Decreased alpha-band power was correlated with loss of consciousness and severity of aversive psychological traumatic stimuli. Decreased alpha band power was seen in mTBI patients more severely affected by PTSD in the superior frontal cortex, rostral middle frontal cortex, caudal middle frontal cortex, and frontal pole.	MEG could be used to detect PTSD associated with mTBI.
[41]	Evaluating the potential of MEG and DTI to detect memory retrieval brain–behavior disconnection in mTBI	OS	23 male mTBI patients (aged 29.9 ± 6.9 years); 18 male HCs (aged 27.3 ± 5.3 years)	Task-related MEG (modified one-back visual working memory task); frequency bands: continuous scan for 1–50 Hz wavelengths; analysis methods: functional connectivity, source localization.	2 weeks–3 months	MEG was able to detect functional disruptions in the brain activity of mTBI patients: limbic–prefrontal circuitry, thalamo-hippocampus process, amygdala activation.	MEG could be used to detect brain functional disruptions in mTBI.
[42]	Exploring the utility of MEG in detecting cognitive impairments in mTBI during cognitive tasks	LS	25 mTBI patients (aged 42 ± 2 years, 56% males, 15 sports injuries, 6 motor vehicles accidents, 4 falls); 20 HCs (aged 39 ± 2 years, 60% males).	Resting-state MEG (1 × 10′ eyes-opened and 1 × 10′ eyes-closed sessions), task-related MEG (paced auditory serial addition test, vigilance test); frequency bands: alpha (8–14 Hz); analysis methods: source modeling of measured oscillatory brain activity.	<6 months	During cognitive tasks, mTBI patients registered decreased alpha band frequencies in task-relevant cortical regions (inferior parietal lobule and frontal cortex). MEG detected significant changes in oscillatory alpha activity in mTBI, as compared to HCs.	MEG could be used to discriminate between mTBI and HCs.
[43]	Exploring the potential of theta-band oscillations as diagnosis biomarkers for mTBI	LS	26 mTBI patients (aged 41 ± 2 years, 57% males); 139 HCs (aged 31 ± 1 years, 26% males)	Resting-state MEG (1 × 10′ eyes opened and 1 × 10′ eyes closed sessions); frequency bands: low-frequency ranges (0.5–7 Hz); analysis methods: functional connectivity, source localization with cortically constrained L2 minimum-norm estimate.	4 weeks and 6–7 months	Structural lesions in mTBI could be correlated with aberrant theta band activity (+ 2 standard deviations from mean).	Theta-band oscillations, as assessed by MEG, could be a valuable biomarker for brain dysfunctions in mTBI.
[44]	Evaluating the potential of machine learning-assisted MEG in detecting mTBI	LS	25 mTBI patients (aged 41 ± 2 years, 57% males); 20 HCs (aged 39 years, 60% males); 621 HCs—as normative data.	Resting-state MEG (1 × 10′ eyes closed sessions); frequency bands: multiple bands; analysis methods: source modeling of measured oscillatory brain activity.	<6 months	Mild TBI patients’ brain oscillatory patterns were characterized by higher average activation at 10, 20, and 30 Hz. Theta-band activity was found to be a significant indicator of mTBI.	MEG is a potent tool in mTBI diagnosis.
[45]	Evaluating the potential of MEG in detecting mTBI-associated injuries	RCT	20 male mTBI patients (aged 31 ± 7 years; 21 male HCs (aged 27 ± 5 years)	Resting-state MEG (1 × 5′ resting session); frequency bands: multiple; analysis methods: functional connectivity, source localization	<3 months	Delta, gamma, and alpha frequency ranges were associated with mTBI injuries. Based on MEG scans, alterations in brain connectivity were correlated with clinical symptom severity.	MEG could be used as a diagnosis tool in mTBI.

MEG—magnetoencephalography; DTI—diffusion tensor imaging; mTBI—mild traumatic brain injury; CT—computer tomography; MRI—magnetic resonance imaging; TIA—time from injury to assessment; OS—observational study; LS—longitudinal study; RCT—randomized controlled study; HCs—age- and sex-matched healthy controls; OTC—orthopedic trauma control; SCAT2—Sport Concussion Assessment Tool 2; PTSD—post traumatic stress disorder.

**Table 2:** Functional connectivity changes in mTBI.

Ref	Outcomes	Conclusion
[27]	Disruption of functional connectivity in default mode network linked to cognitive deficits in mTBI patients.	MEG reveals network alterations that contribute to PCS-related cognitive issues.
[28]	Reduced local efficiency and functional connectivity in mTBI patients.	MEG provides detailed insight into network-level brain communication deficits after mTBI.
[29]	Functional connectivity was found to be affected by mTBI and correlated with cognitive tasks related to perception and motor control.	MEG could be used to detect functional connectivity changes in mTBI.
[31]	Functional connectivity patterns could be used in differentiating mTBI from PTSD.	MEG could be used to detect subtle brain functional connectivity changes in mTBI.
[32]	Low-frequency functional connectivity affected in mTBI.	Low-frequency functional connectivity makers could be used in diagnosing mTBI.
[36]	Brain connectivity was reduced in mTBI, as compared to HCs, when evaluated by task-related MEG.	MEG could be used to detect brain connectivity issues in mTBI.
[39]	Mild TBI disrupts the structural framework underlying neural synchrony and network functions, which are both very subtle consequences of head trauma.	Subtle changes in brain connectivity could be assessed by resting-state and task-related MEG.
[41]	Functional connectivity changes were seen in mTBI.	MEG could be used to detect functional connectivity issues in mTBI.

**Table 3:** MEG for differentiating mTBI from PTSD.

Ref	Outcomes	Conclusion
[28]	Using MEG scans, PTSD was characterized by lower values of network metrics that demonstrate decreased local connectivity and a hierarchically impaired network structure.	Graph-based analysis of resting-state MEG scans could be a valuable tool in diagnosing mTBI and potentially differentiate PTSD from mTBI.
[33]	The regional power from alpha and beta bands could be used as diagnosis markers for mTBI and differentiated diagnosis between mTBI and PTSD, exhibiting the best performance in classification.	MEG could be used to differentiate between mTBI and PTSD.
[40]	Alpha-band power was significantly altered in mTBI patients with severe PTSD.	MEG could be used to detect PTSD associated with mTBI.

**Table 4:** MEG for monitoring recovery in mTBI.

Ref	Outcomes	Conclusion
[26]	Resting-state MEG with machine learning successfully predicted recovery of mTBI patients from PCS at 3 months.	Resting-state MEG could be used to predict recovery from PCS at 3 months.
[34]	The dynamic analysis of brain connectivity over time revealed the decrease in differences between mTBI and OTC.	MEG could be used to assess recovery from mTBI.
[36]	Dynamic network analysis showed microstate-related transitions in mTBI, as compared to HCs.	MEG could be employed to diagnose mTBI by identifying a dynamic connectome biomarker based on functional integration.
[37]	Vision and cognitive deficits were improved by intervention and MEG brain imaging; using the Fast-VESTAL procedure successfully detected that movement discrimination training improved the time-locked activity of several brain networks.	MEG brain imaging could be used to detect cognitive recovery from mTBI.

**Table 5:** Cognitive and behavioral correlates of MEG findings.

Ref	Outcomes	Conclusion
[22]	Functional changes across several brain areas correlated with slower reaction times in the working memory task.	Task-related MEG could evaluate functional deficits correlated with cognitive impairments.
[24]	Greater gamma activity was correlated with poorer cognitive performance.	MEG could be a promising biomarker of mTBI and PCS-associated cognitive impairment.
[27]	Disruption of functional connectivity in default mode network linked to cognitive deficits in mTBI patients.	MEG reveals network alterations that contribute to PCS-related cognitive issues.
[29]	Functional changes across several brain areas correlated with poorer eye movement control in correlation with beta band changes in ten brain regions.	Task-related MEG could evaluate functional deficits correlated with attention and coordination of movement/response.
[32]	Several differences in functional connectivity of the brain were observed in secondary symptoms of mTBI, such as lack of attention, anxiety, and depression.	Functional connectivity changes could reveal the occurrence of overlapping cognitive sequelae in mTBI.
[36]	Altered brain connectivity was correlated with decreased performance in mental flexibility tasks.	A correlation between brain connectivity, as evaluated by MEG, and cognitive performance was established.
[37]	MEG with source localization with fast-VESTAL detected that movement discrimination training improved time-locked activity of several brain networks.	MEG brain imaging could be used to detect cognitive recovery from mTBI.
[38]	The changes in frequency bands were correlated with cognitive symptom severity.	MEG could be used to correlate functional connectivity changes with cognitive task performance.
[41]	Functional and structural disruptions in brain–behavior interactions during working memory tasks were seen in mTBI.	MEG could be used to track brain–behavior interactions.
[42]	Oscillatory alpha activity was correlated with auditory performance.	MEG could be used to correlate cognitive impairments in mTBI patients.

**Table 6:** MEG’s role in detecting subtle brain injury.

Ref	Outcomes	Conclusion
[21]	Subtle neuronal injury detected by MEG, not visible by CT or MRI.	MEG is more sensitive than traditional imaging techniques for diagnosing mTBI.
[33]	The regional power from alpha and beta bands could be used as diagnosis markers for mTBI and differentiated diagnosis between mTBI and PTSD.	MEG could be used to detect subtle brain functional connectivity changes in mTBI.
[38]	MEG was able to detect subtle abnormal brain waves patterns in concussed patients.	MEG could be used to detect subtle brain wave changes.
[39]	Disrupted structural framework underlying neural synchrony and network functions were found in mTBI.	Subtle changes in brain connectivity could be assessed by resting-state and task-related MEG.

**Table 7:** Studies applying machine learning to MEG data for mTBI diagnosis.

Ref	Outcomes	Conclusion
[24]	Machine learning was used to predict the differentiation between mTBI and HCs.	Machine learning could improve diagnosis sensitivity.
[26]	Machine learning was used to complement imaging markers across delta-band and gamma-band frequencies.	Machine learning could improve delta–gamma potential in clinical diagnosis and recovery prediction.
[31]	The machine learning-based downstream data analysis pipeline was used to increase discriminative performance. Machine learning offered more accurate predictions, as compared to standard analysis.	Machine learning could increase the performance of diagnosis procedure.
[33]	Machine learning was used to consistently improve the analysis of subtle brain connectivity changes and discrimination potential. Machine learning performed better in identifying subtle brain connectivity changes and discriminating between mTBI and PTSD, as compared to standard analysis.	MEG could be used to differentiate mTBI from PTSD, with the aid of machine learning.
[39]	Machine learning was used to show that dynamic coordination of neural network activity is impaired in mTBI.	Machine learning could be used in the data processing of MEG scans to detect neural network activity changes specific to mTBI.
[44]	Machine learning could be used to successfully assist MEG to discriminate mTBI patients from HCs.	Machine learning could assist MEG in mTBI diagnosis.

**Table 8:** Limitations of MEG in mTBI diagnosis.

Limitation	Description
Limited accessibility and increased costs	MEG remains a costly and specialized neuroimaging technique, available in only a limited number of clinical centers, posing accessibility restrictions in current clinical practice. The infrastructure and expertise required to conduct and interpret MEG scans can also pose logistical challenges.
Lower spatial resolution, as compared to other imaging techniques	While excelling in temporal resolution, the spatial resolution offered by MEG is inferior to functional MRI, limiting the accuracy of localization of the identified functional abnormalities. Combining MEG with other imaging modalities, such as DTI, may improve diagnostic precision but also adds to complexity and cost.
Study design heterogeneity	The variability of study designs, MEG data-processing methods, and sample sizes posed the most significant challenge. Moreover, the variation in the frequency of the analyzed bands, the conditions in which MEG was administered to the participants (resting-state versus task-based), and the criteria used for patient selection prevented the drawing of uniform conclusions.
Small sample sizes	The generalizability of the findings could be affected by small sample sizes. Larger, multi-centered studies are needed to validate its diagnostic efficacy across diverse populations and injury types.
Lack of standardized MEG protocols	The lack of standardized protocols for MEG data acquisition, analysis, and interpretation in mTBI patients prevents its use as a common evaluation tool in clinical practice. The development of globally accepted guidelines could be essential for clinicians and researchers.

**Table 9:** Future perspectives.

Aspects to Be Developed	Description
Standardized MEG protocols	Establishing standardized MEG protocols for mTBI assessment is crucial for ensuring consistency across studies and enabling wider clinical application. This would involve standardizing data acquisition, pre-processing, and analysis pipelines, as well as defining universally accepted biomarkers for mTBI.
Combining MEG with other neuroimaging techniques	The integration of other imaging techniques, such as DTI or functional MRI, could improve both the spatial and temporal resolution of MEG-based scans. Also, the multimodal approach could improve the sensitivity of detecting structural and functional abnormalities and provide a more comprehensive understanding of mTBI.
Further machine learning and artificial intelligence integration	The analysis of MEG data assisted by machine learning shows promise in improving diagnostic accuracy and predicting clinical outcomes. Further development of AI models that can integrate complex MEG datasets, clinical information, and patient outcomes will enhance the utility of MEG as a predictive tool for mTBI prognosis.
Focusing on longitudinal studies	Longitudinal studies are essential to understand the dynamics of MEG biomarkers and their correlation with clinical recovery. Furthermore, these studies could provide valuable insights about treatment efficiency and rehabilitation strategies, particularly seen in chronic cases of mTBI and PCS.
Cost-effective optimization	Making MEG more cost-effective could be crucial in improving accessibility and diagnostic potential. The costly and non-portable MEG systems and the lack of lower-cost hardware or cloud-based analysis platforms prevents its broader use in clinical settings.
Differentiated diagnosis	The potential of MEG to discriminate between mTBI and other neurological conditions, such as PTSD, was significantly reported. Further research could focus on developing specific MEG-based biomarkers with increased accuracy in distinguishing between overlapping or co-occurring conditions.
